# Balancing motherhood and career: The psychological effects of post-maternity leave return among Tunisian women

**DOI:** 10.1192/j.eurpsy.2025.1602

**Published:** 2025-08-26

**Authors:** A. Karmous, H. Ktari, N. Karmous, H. Ghabi, A. Hajri, E. Khelifa, A. Maamri, H. Zalila

**Affiliations:** 1 Emergency and outpatient department, Razi hospital, Manouba; 2 Psychiatry department, Tahar Maamouri hospital, Nabeul; 3 Department B, Charles Nicoles hospital, Tunis; 4 Department G, Razi hospital, Manouba, Tunisia

## Abstract

**Introduction:**

Tunisia is considered one of the countries where women occupy an important place in the working society. Given the fundamental role played by women in the family, they would have to balance their family responsibilities with professional duties, especially after returning from maternity leave. In many cases, the return to work can be particularly difficult which could affect their physical and mental health and even the relationship with their child.

**Objectives:**

Assessing the effects of returning to work on the perceotion of stress after maternity leave among Tunisian women.

**Methods:**

A cross-sectional study was conducted over a two-month period, from February to April 2024, in the department B of gynaecology and obstetrics in Charles Nicoles hospital (Tunis). Women who were in labor before delivery, who had fully responded to the questionnaire before and after returning to work and willing to take part in the study were included. A questionnaire including socio-demographic, pregnancy and delivery and labor characteristics was filled in by the participants. The effects of returning to work on the perception of stress after maternity leave were assessed using the Perceived Stress Scale (PSS-10). Women were assessed one month and three months after the delivery corresponding to one month after the return to work (maternity leave in Tunisia lasts two months).

**Results:**

A total of 62 women were included. The mean age was 32.45 ± 6.41. Pregnancy was planned in 56.4 % of cases, well supervised in 83.8 % of cases, and complicated by gestational diabetes in 24.1 % of participants. Most deliveries were at full term (92 %) and vaginal (66.1 %). Complications were post-partum hemorrhage (11.2 %), eclampsia (3.2 %) and puerperal infections (3.2 %). Most women worked in the public sector (69.3 %) with a salary between 500 and 1000 Tunisian dinars (42%). The mean PSS-10 before returning to work was 12.36 ± 4.79 and 19.41 ± 4.31 after. After returning to work, the PSS-10 was significantly higher among women with more than two children, whose deliveries were with complications and working in the private sector.

**Conclusions:**

Several factors have been found to involved in the increase of stress after post-maternity leave return. Focus on the psychological aspect and on supporting young mothers throughout the perinatal period seem highly needed.

**Disclosure of Interest:**

None Declared

